# A Comparison between the Antimicrobial Effects of Triple Antibiotic Paste and Calcium Hydroxide Against Entrococcus Faecalis

**Published:** 2012-08-01

**Authors:** Alireza Adl, Nooshin Sadat Shojaee, Mohamad Motamedifar

**Affiliations:** 1. Department of Endodontics, Dental School, Shiraz University of Medical Sciences, shiraz, Iran; 2. Department of Bacteriology and Virology, Medical School, Shiraz HIV/Aids Research Center (SHARC), Shiraz University of Medical Sciences, Shiraz, Iran

**Keywords:** Antibiotics, Antimicrobial Efficacy, Calcium Hydroxide, Entrococcus Faecalis, Intracanal Medicaments

## Abstract

**Introduction:**

The purpose of this study was to determine the in vitro antimicrobial ability against Entrococcus (E.) faecalis of triple antibiotic paste and its components compared with calcium hydroxide mixtures.

**Materials and Methods:**

An agar well diffusion assay and MIC method were used to determine the efficacy of the experimental medicaments in removing E. faecalis. Medicaments were divided into 9 groups; triple antibiotic powder with saline or chlorhexidine, metronidazole, ciprofloxacin, minocycline antibiotics were also separately tested (with normal saline), and Ca(OH)2 (plus normal saline or 2% chlorhexidine). These medicaments were evaluated at four concentrations of 25, 50, 100 and 200 μgram per mL in an agar well diffusion test. The diameters of the growth inhibition zones for each group were recorded and compared. The minimum inhibitory concentrations (MIC) of tested medicaments that are required to kill E. faecalis were also determined. The differences between groups were analyzed by Kruskal-Wallis and Mann-Whitney U tests.

**Results:**

The largest inhibition zones were observed for the triple antibiotic mixture/saline, triple antibiotic mixture/2% chlorhexidine and minocycline/saline, and the smallest for Ca(OH)_2_/saline, Ca(OH)_2_/2% chlorhexidine. Concentration increases produced greater antibacterial effects in all groups. The MIC determination method showed similar results.

**Conclusion:**

The results suggest that the triple antibiotic paste with either 2% chlorhexidine or normal saline would be the preferred medicament against E. faecalis and, among its three components, minocycline has the greatest antibacterial effect.

## Introduction

There have been many studies that prove that bacteria is the major cause of pulpal and periapical diseases [1][2][3][4]. Because of the complex nature of the root canal system and the presence of many inaccessible areas, a combination of mechanical instrumentation and irrigation is necessary to decrease the amount of bacteria/micro-organisms in the root canal system [5]. However, chemo-mechanical preparation is often not enough, and many bacteria may remain in the root canal system [6][7][8].

The use of inter-appointment medicaments may reduce microorganisms that remain in root canals. Ca(OH)_2_ is widely used in endodontics, but this common endodontic medicament does not sufficiently affect Entrococcus (E.) faecalis, the most important bacterium in resistant infections [9][10][11]. E. faecalis is an enteric facultative gram positive bacterium which can grow independently in the root canal without the assistance of other bacteria This microorganism has the ability to invade and live in the dentinal tubules [4][5][6][7][8][9][10][11][12].

Recently, triple antibiotic paste (a mixture of metronidazole, ciprofloxacin and minocycline) has been used as an intracanal medicament for disinfecting the root canal during regenerative procedures [13][14][15][16]. Bose et al., in a retrospective study, showed that Ca(OH)_2_ and triple antibiotic paste, because of their antibacterial properties, can aid further development of the pulp dentin complex when used as an intracanal medicament in immature necrotic teeth [17].

Sato et al. showed triple antibiotic paste can destroy the bacteria in deep areas of the root canal system [18]. In a study on dogs, the results indicated the effectiveness of triple antibiotic paste in the disinfection of immature teeth with apical periodontitis [19]. To date, there is insufficient documentation regarding the antimicrobial effect of triple antibiotic paste in eliminating E. faecalis. The purpose of this in vitro study was to provide further insight into the efficacy of the antibiotic paste in eradicating E. faecalis.

## Materials and methods

### Agar well diffusion assay method

The medicaments tested were in powdered form. They were as follows: the antibiotic mixture, the individual components of the triple antibiotic mixture-metronidazole (Flagyl, Winthrop Pharmaceuticals, UK), ciprofloxacin (Bayer plc, UK) and minocycline (Expanscience Laboratories, Paris, France), Ca(OH)2 powder (Golchay, Tehran, Iran) and talc powder (Royal Minerals, India). The triple antibiotic mixture, Ca(OH)_2_ and talc powder were mixed with either normal saline (NaCl 0.9%, Darupakhsh, Tehran, Iran) or 2% chlorhexidine (Consepsis, Ultradent Inc., South Jordan, UT. USA). Each component of the triple antibiotic paste was just mixed with normal saline. Talc powder mixed with normal saline served as the control group, because a pilot study showed it has no antibacterial activity against E. faecalis (Table 1).

**Table 1:** The details of medicaments tested against E. faecalis

**Group**	**Description**
**1**	Triple antibiotic paste + Normal Saline****
**2**	Triple antibiotic paste + 2% Chlorhexidine****
**3**	Minocycline + Normal Saline****
**4**	Metronidazole + Normal Saline****
**5**	Ciprofloxacin + Normal saline****
**6**	Ca(OH)_2_ + Normal Saline****
**7**	Ca(OH)_2_ + Chlorhexidine 2%****
**8**	Talc powder + Chlorhexidine 2%****
**9**	Talc powder + Normal Saline****

All the medicaments were tested at concentrations of 25, 50, 100 and 200 µgram per mL in normal saline/2% chlorhexidine.

The triple antibiotic paste was prepared for each group with identical amount of the three antibiotic powders (mg) and then mixed with 1 mL normal saline or 2% chlorhexidine.

The bacterium tested was E. faecalis (ATCC 11700). Pure E. faecalis was grown on BHI agar plates (Himedia Laboratories, Mumbai, India). The microorganisms were inoculated into tubes containing 5 mL 0.9% sterile saline solution. The suspension was adjusted by using McFarland tubes to match the turbidity to 1.5×108 cfu/mL. The BHI agar plates were flooded with the test suspension. Then 4 wells (4 mm depth ×6 mm in diameter) were cut in the agar for each concentration of medicaments. A sterile spatula was used to place the pastes into each well.

The plates were then incubated at 37°C under appropriate atmospheric conditions (80% N_2_, 10% CO_2_, 10% H_2_) for 7 days under anaerobic conditions in a CO_2_ incubator (Mart Microbiology B. V., Netherlands). The diameters of the zones of bacterial growth inhibition around the wells containing the test substances were then recorded after the period of incubation. The inhibitory zone was determined in millimeters by measuring the shortest distance between the outer margin of the well and initial microbial growth.

Each experiment was performed six times and the means and standard deviations of the inhibitory zones were calculated. The differences between groups were analysed by Kruskal-Wallis and Mann-Whitney U tests. P values less than 0.05 were considered as significant.

### MIC determination method

A reference bacterial strain E. faecalis (ATCC 11700) obtained from the American type culture collection was used. The bacteria was cultured in Brain heart infusion (BHI agar) culture medium, stored in a tube and incubated at 37°C for 7 days. Microbial cells were resuspended in saline to obtain a final concentration of 1.5×108 cfu/mL, similar to that of tube No. 0.5 on the MacFarland scale. A total of 0.1 mL of each pure suspension was used to obtain a mixture of the test microorganisms.

The experimental medicaments (groups 1–7) were prepared similar to the agar diffusion test. To determine the MIC, six fold dilutions were made for each group.

After dilution of the medicaments, inoculums of 0.09 mL from the experimental suspension were added. These were incubated at 37°C for 7 days.

Microbial growth was evaluated by two methods: turbidity of the culture medium and subculture in a specific nutrient broth. In the latter method, the samples were transferred from tubes with no bacterial growth to nutrient agar plates by means of an inoculating loop, and lack of bacterial growth was confirmed. The lowest concentration at which no bacterial growth was seen was determined to be the MIC of that medicament.

The MIC for each medicament was determined and the experiments were repeated four times. Then the mean values and standard deviations were calculated. Comparisons were made among the MIC values of different medicaments using the Kruskal-Wallis test and Mann-Whitney as post-hoc test.

## Results

### Agar well diffusion assay method

The means and standard deviations of the diameters of the growth inhibition zones for each concentration of the preparations are presented in Table 2.

**Table 2:** The means (SD) of agar well diffusion assay-(mm) after 7 days from 9 separate groups

**Group/concentration**	**25 µg**	**50 µg**	**100 µg**	**200 µg**
**Group 1**	33.83 (0.40) a	35.66 (0.51)^a^	36.50 (0.54)^a^	38.16 (0.75)^a^
**Group 2**	35.66 (0.56)^a^	36.66 (0.51)^a^	37.50 (1.24)^a^	39.66 (1.03) a
**Group 3**	34.83 (0.75)^a^	35.83 (0.75)^a^	37.83 (0.75)^a^	39.33 (0.81)^a^
**Group 4**	18.83 (0.75)^a^	20.83 (0.75)^a^	26.16 (0.93)^a^	29.50 (0.54)^a^
**Group 5**	26.66 (1.50)^a^	28.66 (0.51)^a^	29.83 (0.75)^a^	34.00 (0.63)^a^
**Group6**	0.00 (0.00)	0.00 (0.00)	4.33 (4.75)	9.00 (4.47)^a^
**Group7**	0.00 (0.00)	0.00 (0.00)	6.66 (5.16)^a^	11.66 (1.03)^a^
**Group8**	0.00 (0.00)	0.00 (0.00)	1.66 (4.08)	6.50 (5.04)
**Group9**	0.00 (0.00)	0.00 (0.00)	0.00 (0.00)	0.00 (0.00)

^a^ Indicates a significant difference (P<0.05) compared with the control group (group 9) according to Mann-Withney U test

The range of inhibitory values between experimental groups varied broadly and showed significant differences (P<0.05).

**Figure 1:**
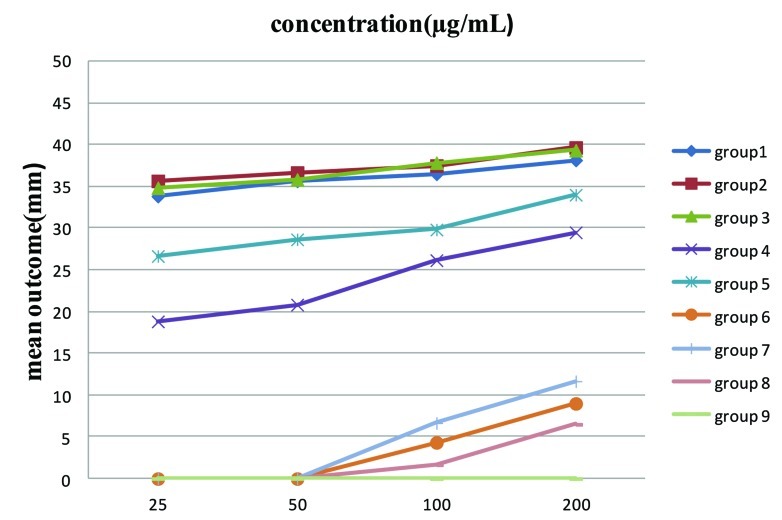
Comparison of groups1-9 in different concentrations

Each experimental group was compared to the talc/saline control (group 9) separately. Overall, triple antibiotic paste/saline, triple antibiotic paste/2% chlorhexidine and minocycline/saline (groups 1, 2, 3) had the largest zones of growth inhibition in the well diffusion assay at all concentrations (Figure 1). Metronidazole and ciprofloxacin (groups 4, 5) had significant inhibitory effects on E. faecalis at all concentrations compared to the control group (P<0.05).

Ca(OH)_2_/saline was effective against E. faecalis only at 200 µgm/mL and Ca(OH)_2_/2% chlorhexidine showed inhibitory effect even at concentrations of 100 and 200 µgm/mL (P<0.05).

Talc/2% chlorhexidine showed minimal inhibitory effects at concentrations of 100 and 200 µgm/mL, which were not significantly different from the control group (P<0.05).

Increasing the concentrations of the tested medicaments produced inhibition zones with larger diameters in all groups, (P<0.05); i.e they had greater inhibitory effect on E. faecalis.

### MIC determination method

The minimum inhibitory concentration (MIC) was defined as the lowest concentration able to inhibit any visible bacterial growth.

MIC’s of the tested medicaments required to kill the bacterium are shown in Table 3.

**Table 3:** The means (SD) of MICs of tested medicaments

**Group**	**MIC(µg/mL)**
**Group 1**	77.5(22.50)****
**Group 2**	325(225.00)****
**Group 3**	325(225.00)****
**Group 4**	550(259.80)****
**Group 5**	3250(2250.00)****
**Group 6**	**-----------------**
**Group 7**	195000(135000.00)****

The most effective medicament against E. faecalis was triple antibiotic powder/normal saline, with a MIC equal to 77.5 µgram per mL. Triple antibiotic powder/ 2% chlorhexidine and minocycline/normal saline had similar MIC values, equivalent to 325 µgram per mL.

The least effective was group 7 (Ca(OH)_2_ /2% chlorhexidine), which had a MIC equal to 195000 µgram per mL. The results also showedthat group 6 (Ca(OH)_2_+normal saline) was not active against E. faecalis even at the highest concentration used in this study.

Statistical analysis showed a significant difference between group 7 [Ca(OH)_2_/2% chlorhexidine 2%] and the other medicaments. Also, group 5 (ciprofloxacin/normal saline) was significantly less effective than groups 1-4.

## Discussion

The agar diffusion test we used in this study is useful for evaluating and comparing the in vitro antimicrobial activities of medicaments before performing more advanced tests; many studies have used this method for evaluations of antibacterial effects of various endodontic materials.[20][21][22]. The results obtained from this test must be interpreted with caution, as this assay may not demonstrate the full clinical potential of the material being tested. The MIC test is also a research tool to determine the in vitro activity of new medicaments and antimicrobials. Asna ashari et al. used two techniques, including zone of inhibition (ZI) and minimum inhibitory concentration (MIC), to evaluate the antimicrobial effects of MTAD, sodium hypochlorite (NaOCl) and their combination on endodontic microorganisms. Similarly, in the current study we used these methods to compare the antimicrobial effects of intracanal medicaments [22].

The bacterial species E. faecalis was selected as representing an organism commonly isolated from the root canals of teeth that have been previously root filled [23]. There have been several studies which applied E. faecalis as a target microorganism to evaluate the effects of antibacterial agents [24][25][26]. In fact, previously treated failed teeth are nine times more likely to be infected with E. faecalis than primary endodontic infections [27]. E. faecalis is also resistant to calcium hydroxide, a commonly used intracanal medicament, especially when a high pH is not maintained [28][29][30].

Some studies have shown promising effects of chlorhexidine in a 2% gel or liquid form to reduce or completely eliminate E. faecalis from the root canal space and dentinal tubules [31][32][33]. Turk et al. showed calcium hydroxide mixed with 2% chlorhexidine digluconate was more effective against E. faecalis than calcium hydroxide mixed with other vehicles [34]. Therefore we utilised chlorhexidine as one of the vehicles for this study. To ascertain which substance had the highest antibacterial effect, each component was independently mixed with normal saline. The results showed the triple antibiotic powder, either mixed with normal saline or 2% chlorhexidine, produced the largest zone of inhibition against E. faecalis. The minocycline/normal saline combination was very effective activity against E. faecalis. Although metronidazole/normal saline and ciprofloxacin/normal saline showed antibacterial efficacies significantly higher than the control group, their ability to kill E. faecalis was less than minocycline and the triple antibiotic paste, according to both the agar diffusion and MIC tests. It can be concluded that minocycline is the most effective component of the triple antibiotic paste against E. faecalis, as adding the two other antibiotics to the mixture did not considerably increase the diameter of its zones of inhibition.

Pinheiro et al. reported most of the 21 microbial isolates from root canals of filled teeth with persistent periapical lesions were susceptible to tetracycline and doxycycline, which is supports the outcome of the present study that showed minocycline is the most effective component against E. faecalis [35]. Antibacterial activity of chlorhexidine may be attributed to its chemical characteristics.

In the present study, the antimicrobial effect of the triple antibiotic paste and its three components against E. faecalis were compared to calcium hydroxide, either mixed with normal saline or 2% chlorhexidine. Considering the low antibacterial capability of talc powder/chlorhexidine and Ca(OH)_2_ chlorhexidine, and the high ability of triple antibiotic powder/normal saline in this study, we conclude the ability of the former is related to its mixed antibiotic contents, not to the vehicle’s properties.

The results of the present study also indicated that calcium hydroxide in normal saline or 2% chlorhexidine had some antibacterial efficacy against E. faecalis, but only at concentrations of 100 and 200 µgram per mL. The zones of inhibition generated by groups containing calcium hydroxide were much smaller than the zones of the antibiotic-containing groups, concurring with previous literature that calcium hydroxide is not an effective intracanal medicament when root canals are infected by E. Faecalis [28][29][30].

The study showed that the higher the concentrations (25 µgram per mL to 200 µgram per mL) of antibiotics or calcium hydroxide, the greater its' antibacterial efficacy. A similar trend was reported by Blanseet et al. for different mixtures of calcium hydroxide [36].

E. faecalis is the most frequent species in failed root canal therapy cases [9][10][11]; therefore the findings of the present study support the use of triple antibiotic paste as a potential intracanal medicament in the treatment of endodontic failures. It is noteworthy to mention that triple antibiotic paste is utilised in primary infections of open apex teeth, in which E. faecalis is not a prevalent bacteria [13][14][15][37].

In vitro tests may not show the full clinical potential of a tested material, as dentin can interact with endodontic disinfectants [38]. We suggest future investigations repeat the efficacy of the triple antibiotic paste in reducing CFUs in a dentin diffusion model, using clinically relevant periods of disinfection and deep testing of dentin.

## Conclusions

Under the limitations of this study, the triple antibiotic paste is very effective against E. faecalis and can be considered as a more powerful root canal medicament compared to calcium hydroxide pastes. The results also show that minocycline is the most effective component of the triple antibiotic paste.
